# The Effect of Behavioral Activation on Older Adults' Engagement and Wellbeing: A Randomized Control Trial Protocol

**DOI:** 10.1093/geroni/igab046.3279

**Published:** 2021-12-17

**Authors:** Julia Turner Scott, Mary Luszcz, Trevor Mazzucchelli, Ruth Walker, Tim Windsor

**Affiliations:** 1 Flinders University, Brompton, South Australia, Australia; 2 Flinders University, Adelaide, South Australia, Australia; 3 Curtin University, Perth, Western Australia, Australia

## Abstract

Meaningful activity engagement in later life is widely recognized as crucial for ageing well, but age-related changes and transitions can impede such participation. A behavioral activation framework can provide a person-centred, value-consistent therapeutic approach to increasing activity engagement that is both easy to administer, cost effective and accessible to a broad audience. Although there is evidence supporting the utility of behavioral activation as a treatment for depression in older adults, this study will be the first to examine whether a behavioral activation is more effective in increasing activity engagement and psychological wellbeing among a non-clinical sample of older adults, compared to a multi component positive psychology intervention. This randomized controlled trial will examine the impact of two therapeutic approaches on activity engagement and wellbeing among older adults. One hundred and fifty adults aged 65+ who have relatively lower scores on a measure of engagement with life will be randomized to either a behavioral activation-based intervention, or a multi-component positive psychology intervention. The interventions will involve six individual weekly sessions conducted via telephone or video conference. Participants will be assessed pre-, post-intervention, and at three months follow-up. Outcome measures will include activity engagement, positive affect, and psychological wellbeing. Intra-individual variability will also be assessed via micro-longitudinal data in the behavioral activation condition. This study will be the first to provide evidence to the effectiveness of behavioral activation as an intervention to increase activity engagement and wellbeing among older adults, compared to other therapeutic approaches to increase psychological wellbeing.

